# Two Novel Semi-Quantum Secure Direct Communication Protocols in IoT

**DOI:** 10.3390/s24247990

**Published:** 2024-12-14

**Authors:** Yuan Tian, Nanyijia Zhang, Jian Li

**Affiliations:** 1 College of Information and Control Engineering, Xi’an University of Architecture and Technology, Xi’an 710055, China; 2 School of Cyberspace Security, Beijing University of Post and Telecommunications, Beijing 100876, China

**Keywords:** IoT security, semi-quantum cryptography, semi-quantum secure direct communication, single photons

## Abstract

As Internet of Things (IoT) technology continues to advance, there is a growing awareness of IoT security within the industry. Quantum communication technology can potentially significantly improve the communication security of IoT devices. Based on semi-quantum cryptography and utilizing single photons, this paper introduces two semi-quantum secure direct communication (SQSDC) protocols for use in smart door locks. Protocol 1 is more efficient, and the efficiency analysis shows that the communication efficiency is as high as 28.57%. Security analysis demonstrates the asymptotic security of the protocols, effectively resisting intercept–measure–resend attacks and entangle–measure attacks from potential eavesdroppers. The extended SQSDC protocol (protocol 2) builds upon protocol 1 by enabling a single qubit to transmit two bits of information, resulting in a double efficiency outcome.

## 1. Introduction

The rapid advancement and extensive utilization of the Internet of Things have introduced novel challenges and opportunities within the communication sphere. The IoT connects a variety of smart devices through Internet technology, facilitating the exchange and interaction of information. This is particularly evident in the field of smart homes, where smart door locks have become a common feature of many people’s lives. These smart door locks are connected through the Internet and support a variety of convenient functions such as remote control, fingerprint recognition, and password input, thereby greatly improving security and convenience. However, as technology advances, smart door locks are also facing increasingly serious security challenges.

In the contemporary era, a considerable number of manufacturers of intelligent door locks are utilizing cloud service platforms as intermediaries with the objective of optimizing the user experience and enhancing system flexibility. Upon initiation of a remote control request via a mobile application, for example, by pressing the “unlock” button, the command is transmitted to the cloud platform via the Internet. Upon receipt of the request by the cloud platform, the user’s identity is verified (e.g., login information or password). Thereafter, the unlocking command is transmitted to the door lock. The door lock, in turn, receives the command from the cloud platform via the Internet and performs the corresponding operation, such as unlocking or locking the door. It is possible for hackers to bypass the security mechanisms of door locks through cyber-attacks or password cracking, which can result in the leakage of device data privacy. In this process, the confidentiality and protection of information transmission is a major challenge for smart door locks.

In order to enhance security, it is possible to combine quantum and classical communications in a way that provides a more solid guarantee. The confidentiality of user information and command transmission are the domain of quantum communication, while classical communication is responsible for tasks such as authentication and door lock control. This architectural approach allows classical and quantum systems to complement each other’s strengths. The fundamental benefit of quantum communication is its “unconditional security,” which is guaranteed by the quantum unclonability theorem. This ensures that information cannot be copied or stolen by a third party during transmission, significantly enhancing privacy. Despite the complex transmission and control involved in communication between the cloud platform and the smart door lock, the addition of quantum communication can effectively prevent the content of the communication from being intercepted or tampered with. Quantum communication technology offers innovative solutions to IoT security issues and application scenarios [1,2,3]. For example, in 2021, Al-Mohammed et al. [1] proposed a new approach for simulating the quantum key distribution between IoT devices and a server to encrypt the data sent to the server. In 2023, Fu et al. [2] proposed a scheme of controlled remote quantum state preparation and quantum teleportation based on multiple communication parties, and a nine-qubit entanglement channel was used to achieve secure communication of multiple devices in the IoT. In 2024, Shi et al. [3] proposed a novel quantum scheme for privacy-preserving range MAX/MIN query in edge-based Internet of Things by using OSID quantum protocols. Compared with the related classical schemes, our proposed quantum scheme has higher security.

Since the emergence of Shor’s [4] and Grover’s [5] algorithms, interest in quantum computing and quantum information has steadily increased. Grover’s search algorithm achieves a square root speed-up in unordered searches compared to classical algorithms, while Shor’s algorithm solves the problems of integer factorization and discrete logarithms at an exponential rate. These two problems form the foundation of classical public-key cryptography and present a valuable opportunity for research in quantum cryptography.

In recent years, there have been significant advancements in quantum information science, with increasing attention on quantum cryptography. In 1984, Bennett and Brassard [6] introduced the first quantum key distribution protocol (BB84 protocol), which is based on the principles of quantum mechanics. This breakthrough greatly advanced the field of quantum cryptography. In order to address the various security issues, scholars have put forward a number of different protocols based on quantum cryptography, which have been designed for use in different application scenarios. Including quantum secret sharing (QSS) [7,8], quantum key distribution (QKD) [9,10], quantum private comparison (QPC) [11,12], quantum signature (QS) [13], secure quantum summation [14], quantum secure direct communication (QSDC) [15,16,17,18,19], etc.

The first semi-quantum key distribution (SQKD) protocol was proposed by Boyer et al. in 2007 [20]. The concept of semi-quantum implies that the sender is a fully quantum communicator, while the receiver has limited quantum capabilities, being able to prepare qubits in {$|0\rangle$, $|1\rangle$} states using the Z-basis, measure qubits in the Z-basis, reorder qubits through different delay lines, and send or return qubits. After the introduction of the semi-quantum concept, several semi-quantum protocols emerged, including semi-quantum secret sharing (SQSS) [21], semi-quantum private comparison (SQPC) [22,23], semi-quantum key distribution (SQKD) [24,25], semi-quantum secure direct communication (SQSDC) [26,27,28,29,30,31,32,33,34,35,36,37,38], etc.

The QSDC protocol facilitates direct information transmission between two participants via a quantum channel, without the need to preshare a secret key. In 2002, Long and Liu [15] proposed the initial QSDC protocol, which also introduced a two-step communication strategy for the first time. In 2003, Deng et al. [16] introduced a two-step QSDC protocol using blocks of EPR pairs that was based on the concepts presented by Long and Liu. The scheme is secure to the extent that it is not possible for an eavesdropper to obtain both sequences simultaneously. Subsequently, numerous QSDC protocols have been proposed by scholars [17,18,19]. For example, in 2020, Pan et al. [18] reported on an experimental implementation of free-space quantum secure direct communication based on single photons. In 2022, Sheng et al. [19] proposed a one-step, secure, direct quantum communication protocol, which only requires the distribution of polarization-spatial-mode hyperentanglement for one round.

However, QSDC protocols require the costly utilization of quantum resources and sophisticated technical requirements. In contrast, SQSDC reduces the necessity for quantum resources, simplifies the implementation process, and consequently lowers costs and technological thresholds. In 2014, Zou and Qiu et al. [26] introduced a three-step SQSDC protocol based on single photons that allows a classical participant lacking a quantum register to securely transmit their secret information to a quantum participant. In 2017, Gu et al. [27] identified a novel attack, the double CNOT attack, against Zou and Qiu’s SQSDC protocol. This attack enables an eavesdropper to obtain secret information through a double C-NOT attack. To address this problem, an improvement to the scheme was proposed. In 2019, Wang et al. [28] proposed a two-step SQSDC protocol based on single photons with identity authentication, which can be used to prevent impersonation and the man-in-the-middle attack. In 2020, Rong and Qiu et al. [29] proposed two new SQSDC protocols based on single photons to study how to simultaneously reduce the quantum resource requirements of both quantum and classical participants. The first protocol is more efficient than the second, while the second can save more quantum resources. It is observed that the SQSDC protocols referenced above are all based on single photons, whereas the majority of other protocols are based on either Bell states or GHZ states. To illustrate, in 2020, Rong et al. [30] put forth an SQSDC protocol that employs Bell states and can be extended to multi-party communication using N-particle GHZ states. This protocol has been demonstrated to be fully robust. In 2022, Yang et al. [31] proposed multi-party communication based on GHZ states, enabling one quantum user to communicate with two classical users. In 2023, Guo et al. [32] proposed a multi-party SQSDC protocol based on GHZ states to realize two-way communication between quantum and classical users. In addition, Xu et al. [33] introduced a multiparty SQSDC protocol using four-particle cluster states, leading to higher qubit efficiency than previous SQSDC protocols. In 2024, Tian et al. [34] proposed two SQSDC protocols utilizing the W-state.

The present paper puts forth two single-photon-based SQSDC protocols for IoT smart door locks. These protocols are designed to improve security while conserving quantum resources. The protocol based on single photons is more straightforward and easier to implement physically. In both protocols, the classical participant does not require the use of quantum memory or quantum delay lines. The protocol 1, in which the quantum user is required to prepare only single states for transmission to the classical user, allows the preparation of fewer qubits for the transmission of a greater quantity of secret information than is currently possible with existing protocols. Subsequently, we enhance the efficiency of information transmission. In the extended protocol, which is based on protocol 1, one qubit is used to transmit two bits of secret information, thus achieving a doubling efficiency.

The rest of the paper is organized as follows. Section 2 describes the steps of protocol 1 based on a single photon and gives examples of the protocols. Section 3 presents the security analysis of the two protocols. Section 4 compares the existing single-photon-based SQSDC protocols and gives a brief summary. Section 5 discusses protocol 2 the extended protocols of protocol 1. Section 6 summarizes the paper.

## 2. Protocol Design

In this paper, we propose a single-photon-based SQSDC protocol for smart door lock scenarios in the IoT. The protocol does not require classical participants to use quantum memory or quantum delay lines. Below are the concrete steps of this protocol, and we consider the above communication scenario modelled with the quantum participant Alice (smart door lock) and the classical participant Bob (user).

### 2.1. Proposed Protocol (Protocol 1)

1. In this paper, we propose a single-state SQSDC protocol in which quantum Alice only needs to prepare 2n single photons $|+\rangle$ and sends them to Bob.
2. Once Bob has received all the qubits, the n particles are randomly selected from them to form the sequence $S_{1}$, while the remaining particles constitute the sequence $S_{2}$. For sequence $S_{1}$, Bob randomly selects either the CTRL operation (which returns the particle to its original state without any additional processing) or the SIFT operation (which measures the particle with the Z-basis, records the measurement, and returns the particle to its original state). For sequence $S_{2}$, Bob performs the re-prepare operation (which directly discards the particle and re-prepares a qubit) and message encoding with the following encoding rules: Bob prepares the secret messages $M=\{m_{1},m_{2},...,m_{n}\}$ and produces code messages $S_{E}=M⊕1=\left\{m_{1}⊕1,m_{2}⊕1,...,m_{n}⊕1\right\}$. Then, $S_{E}$ is encoded into code sequence $S_{E}^{\prime }$ in the Z-basis and sent to Alice. If Bob wants to transmit the message “0”, he would effectively discard the original particle and prepare a state $|1\rangle$ to return to Alice. If Bob wants to transmit the message “1”, he would have to effectively discard the original particle and prepare a state $|0\rangle$ to return to Alice.
  Ultimately, the 2n particles are returned to Alice.
3. For all qubits arriving, Alice temporarily stores them all in the quantum memory and transmits an acknowledgement message to Bob, who then announces the positions of the particles in sequence $S_{1}$, the operation performed on the particles and the measurement results of the SIFT operation. Alice receives and performs eavesdropping detection on the different operations performed by Bob. For particles where Bob performs a CTRL operation, Alice measures them directly with an X-basis, and the result must be $|+\rangle$. The particle selected by Bob for the SIFT operation is directly measured using the Z-basis, and the resulting measurements are then compared with Bob’s own measurements, which should be identical. Alice performs eavesdropping detection based on the results of the above measurements. If the error rate of the detection is higher than a threshold, the communication is abandoned and restarted; otherwise, the process continues to the next step.
4. If there is no eavesdropping, Alice performs the decoding of the message. Alice measures the qubit in the code sequence $S_{E}^{\prime }$, and the results are indicated as $S_{E}^{\prime \prime }$. The decoded messages are $S_{D}\;=\;S_{E}^{\prime \prime }⊕1\;=\;S_{E}⊕1\;=\;M⊕1⊕1\;=M$.

**Figure 1:**
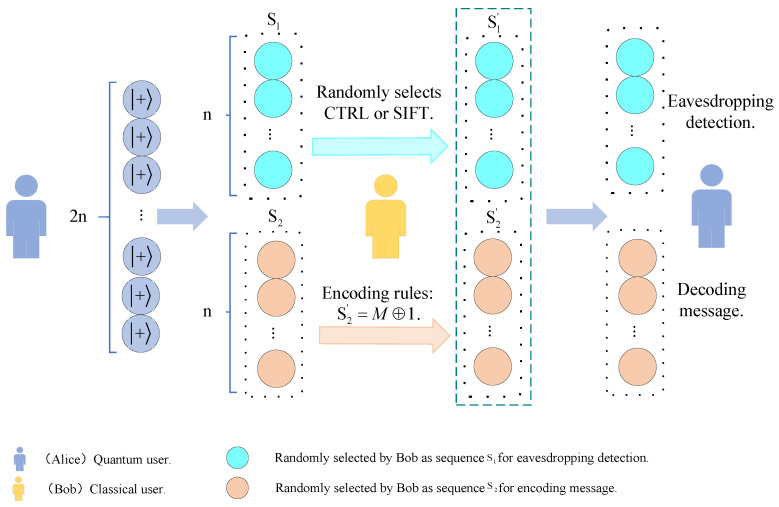
The working of the proposed protocol.

**Figure 2:**
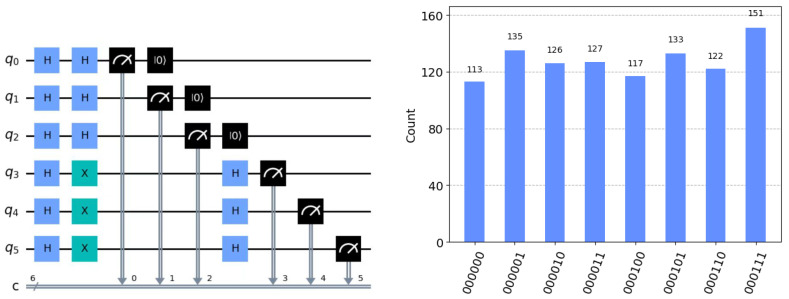
The first picture represents the protocol’s circuit simulation diagram. Applying a Hadamard (H) gate will put them into superposition states. The X gate represents a qubit flip operation, and the black arrow means measurement. The second picture represents the measurement outcomes for the simulation.

Figure 1 shows the flowchart of the proposed protocol. Figure 2 shows the simulated circuit diagram of the communication between Alice and Bob and the measurement results of Alice.

### 2.2. Example of Proposed Protocol

**Figure 3:**
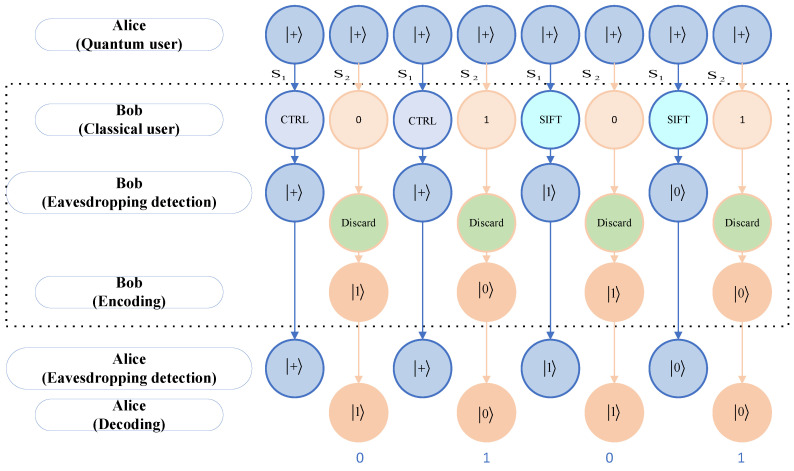
The flowchart of the example.

The steps of the example are described below, and Figure 3 shows a flowchart of the example.

1. Assume that Alice prepares 8 states $|+\rangle$ and transmits them to Bob.
2. Bob receives them and randomly selects 4 for the sequence $S_{1}$. Suppose Bob selects the 1st, 3rd, 5th, and 7th particles for the sequence $S_{1}$. Then, the remaining 2nd, 4th, 6th, and 8th particles are sequence $S_{2}$. In this context, it is assumed that Bob performs the operation {CTRL, CTRL, SIFT, SIFT} on the sequence $S_{1}$ for the purpose of eavesdropping detection. If we assume that Bob wishes to transmit the secret message M={0,1,0,1}, four particles of sequence $S_{2}$ are directly discarded. The code message $S_{E}=M⊕1=\left\{0⊕1,1⊕1,0⊕1,1⊕1\right\}=\left\{1,0,1,0\right\}$. So, Bob returns {$|1\rangle$, $|0\rangle$, $|1\rangle$, $|0\rangle$}.
3. Alice receives 8 particles and sends an acknowledgement to Bob, who tells Alice the location of the particles of sequence $S_{1}$, and the operation performed on the particles, as well as the measurements of the SIFT operation performed, and Alice performs the eavesdropping detection. For particles with direct CTRL operations, Alice measures them directly with an X-basis, and the result should be $|+\rangle$. For the particles that were selected for the SIFT operation, Alice conducts measurements using the Z-basis and compares the results with those obtained by Bob, which were expected to be identical. If there are no errors in the eavesdropping detection, the process proceeds to the next step. Otherwise, it is necessary to repeat the initial step.
4. If there is no eavesdropping, Alice directly measures remaining particles with the Z-basis, which results in {$|1\rangle$, $|0\rangle$, $|1\rangle$, $|0\rangle$}.
  So, Alice receives the message $S_{D}=\left\{1⊕1,0⊕1,1⊕1,0⊕1\right\}=\left\{0,1,0,1\right\}$ transmitted by Bob.

## 3. Security Analysis

### 3.1. Trojan Horse Attack

Eve, an eavesdropper, tries to carry out a Trojan horse attack on the sequences to gain advantageous information. However, this attack can be effectively countered by using photon number splitters and optical wavelength filters [39,40].

### 3.2. Intercept–Measure–Resend Attacks

An intercept–measure–resend attack is a form of eavesdropping whereby the eavesdropper, Eve, intercepts the sequences S that are sent from Alice to Bob and then forwards to Bob the sequences that she has already measured. Should Eve attempt to obtain secret information through the use of an intercept–measure–resend attack, she will inevitably be detected. Without loss of generality, if Eve intercepts the sequences S and chooses to use X-basis measurements, then the single-photon $|+\rangle$ measurement results in $|+\rangle$. Eve will send it to Bob, and whether Bob chooses CTRL or SIFT, an error is not introduced; thus, Eve evades eavesdropping detection. If Eve chooses to use a Z-basis measurement, then the single-photon measurement results in $|0\rangle$ or $|1\rangle$. This photon state will then be transmitted to Bob by Eve. Bob randomly chooses either the CTRL operation or the SIFT operation. If Bob chooses the CTRL operation, then Alice will identify the error and thereby ascertain the presence of the eavesdropper. Conversely, if Bob selects the SIFT operation, the eavesdropper will evade detection. The probability of a particle chosen by Eve for eavesdropping detection is $\frac{1}{2}$, and the probability of a random choice for measurement with an X-basis or Z-basis is also $\frac{1}{2}$. When Eve chooses to measure with the X-basis, no error is introduced whether Bob chooses the CTRL operation or the SIFT operation. When Eve chooses to measure with the Z-basis, the probability that Bob will select either the CTRL or SIFT operation is $\frac{1}{2}$. When Bob chooses to process with the SIFT operation, no error is introduced; however, When Bob chooses to process with the CTRL operation, Alice measures with the X-basis, which detects the error, and thereby discovers the eavesdropping. Therefore, the eavesdropper Eve can evade detection by Alice with a probability of $p=\frac{1}{2}\times \left(\frac{1}{2}\times 1+\frac{1}{2}\times \frac{1}{2}\right)=\frac{3}{8}$ in the intercept–measure–resend attack.

### 3.3. Entangle–Measure Attack

In the entangle–measure attack, an outsider eavesdropper, designated as Eve, intercepts the sequence transmitted from the preceding participant during the quantum sequence’s transmission. Subsequently, she performs unitary operations to entangle the prepared auxiliary particle sequence, designated as $E=\{|E_{0}\rangle ,|E_{1}\rangle ,\cdots ,|E_{n}\rangle \}$, with the intercepted single-photon sequence. The unitary operations performed on each single photon can be represented as follows:

(1) $$U|E_{i}\rangle |0\rangle =|e_{00}\rangle |0\rangle +|e_{01}\rangle |1\rangle ;$$

(2) $$U|E_{i}\rangle |1\rangle =|e_{10}\rangle |0\rangle +|e_{11}\rangle |1\rangle ;$$

(3) $$\begin{array}{cc} \; & U|E_{i}\rangle |+\rangle =U|E_{i}\rangle ⊗\frac{|0\rangle +|1\rangle }{\sqrt{2}}\\ \; & =\frac{1}{\sqrt{2}}(|e_{00}\rangle \left|0\rangle +\right|e_{01}\rangle |1\rangle +|e_{10}\rangle |0\rangle +|e_{11}\rangle |1\rangle )\\ \; & =\frac{1}{\sqrt{2}}\left(|e_{00}\rangle ⊗\frac{|+\rangle +|-\rangle }{\sqrt{2}}+|e_{01}\rangle ⊗\frac{|+\rangle -|-\rangle }{\sqrt{2}}+|e_{10}\rangle ⊗\frac{|+\rangle +|-\rangle }{\sqrt{2}}+|e_{11}\rangle ⊗\frac{|+\rangle -|-\rangle }{\sqrt{2}}\right)\\ \; & =\frac{1}{2}[|+\rangle (|e_{00}\rangle +|e_{01}\rangle +|e_{10}\rangle +|e_{11}\rangle )+|-\rangle (|e_{00}\rangle -|e_{01}\rangle +|e_{10}\rangle -|e_{11}\rangle )]; \end{array}$$

(4) $$\begin{array}{cc} \; & U|E_{i}\rangle |-\rangle =U|E_{i}\rangle ⊗\frac{|0\rangle -|1\rangle }{\sqrt{2}}\\ \; & =\frac{1}{\sqrt{2}}(|e_{00}\rangle \left|0\rangle +\right|e_{01}\rangle |1\rangle -|e_{10}\rangle |0\rangle -|e_{11}\rangle |1\rangle )\\ \; & =\frac{1}{\sqrt{2}}\left(|e_{00}\rangle ⊗\frac{|+\rangle +|-\rangle }{\sqrt{2}}+|e_{01}\rangle ⊗\frac{|+\rangle -|-\rangle }{\sqrt{2}}-|e_{10}\rangle ⊗\frac{|+\rangle +|-\rangle }{\sqrt{2}}-|e_{11}\rangle ⊗\frac{|+\rangle -|-\rangle }{\sqrt{2}}\right)\\ \; & =\frac{1}{2}[|+\rangle (|e_{00}\rangle +|e_{01}\rangle -|e_{10}\rangle -|e_{11}\rangle )+|-\rangle (|e_{00}\rangle -|e_{01}\rangle -|e_{10}\rangle +|e_{11}\rangle )]. \end{array}$$

$\{|e_{00}\rangle ,|e_{01}\rangle ,|e_{10}\rangle ,|e_{11}\rangle \}$ are four pure quantum states that are determined by the unitary operations U and satisfy the following condition.

(5) $$\sum_{\alpha ,\beta }\langle e_{\alpha ,\beta }|e_{\alpha ,\beta }\rangle =1$$

In the proposed protocol, the eavesdropping detection is performed between each transmission of the quantum sequence. If the decoy photon is in state $|0\rangle$ or $|1\rangle$ and Eve wants to avoid detection, the parameters b and c must satisfy b = c = 0. Similarly, if the decoy photon is in state $|+\rangle$ or $|-\rangle$ and Eve wants to avoid detection, then $|e_{00}\rangle -|e_{01}\rangle +|e_{10}\rangle -|e_{11}\rangle =\vec{0}$ and $|e_{00}\rangle +|e_{01}\rangle -|e_{10}\rangle -|e_{11}\rangle =\vec{0}$.

Therefore, we can easily deduce that

(6) $$|e_{00}\rangle =|e_{11}\rangle ;$$

(7) $$U|E_{i}\rangle |0\rangle =|e_{00}\rangle |0\rangle ;$$

(8) $$U|E_{i}\rangle |1\rangle =|e_{00}\rangle |1\rangle ;$$

(9) $$U|E_{i}\rangle |+\rangle =|e_{00}\rangle |+\rangle ;$$

(10) $$U|E_{i}\rangle |-\rangle =|e_{00}\rangle |-\rangle .$$

It is evident that the auxiliary particles are not associated with the intercepted ones. Regardless of the intercepted particles, the auxiliary particles will invariably be in ∣$e_{00}\rangle$. Consequently, Eve will be unable to evade eavesdropping detection by performing the entangled-measure attack, and her attempts to obtain any valuable information regarding Alice’s or Bob’s private inputs will be unsuccessful.

## 4. Comparison

This equation is used to calculate the communication efficiency of the proposed protocol.

(11) $$\eta =\frac{b_{s}}{q_{t}+q_{s}},$$

where $b_{s}$ is the number of bits of actual information received, $q_{t}$ is the number of initial qubits prepared, and $q_{s}$ is the number of classical bits newly prepared later.

In this protocol, Alice prepares 2n $|+\rangle$ states and sends them to Bob. When Bob receives them, he randomly selects n particles to form the $S_{1}$ sequence and the remaining particles to form the $S_{2}$ sequence. For the $S_{1}$ sequence, Bob randomly chooses the CTRL or SIFT operation. For particles with the CTRL operation, Bob only needs to return directly without preparing new particles; for particles with the SIFT operation, Bob needs to prepare a particle of the same state and return it with Z-basis measurement. Thus, in the $S_{1}$ sequence, the number of new particles to be prepared is approximately $\frac{n}{2}$. For the $S_{2}$ sequence, which Bob uses to deliver the secret message, new particles need to be prepared. In order to transmit the secret message “0”, it is necessary to discard the particle and then prepare a new particle to return in state $|1\rangle$. Similarly, to transmit the message “1”, the particle must be discarded, and a new particle prepared to return in state $|0\rangle$. Therefore, the number of new particles that need to be prepared in the $S_{2}$ sequence is n.

The efficiency is:

(12) $$\eta =\frac{n}{2n+\frac{n}{2}+n}=\frac{2n}{7n}=28.57\%.$$

Table 1 presents a comparative analysis of the efficiency of SQSDC protocols based on single photons. The data demonstrate that our proposed protocol exhibits superior efficiency.

Table 2 presents a comparison between the proposed protocol and existing protocols. The majority of current protocols utilize Bell and GHZ states, with fewer employing single photons. Consequently, there is a need to study single-photon-based SQSDC protocols. Our proposed protocol demonstrates superior efficiency compared to previous ones.

## 5. Protocol Extension

We find that the proposed protocol has good extensibility and is able to increase the transmission capacity of a single qubit to the point where two bits of information can be transmitted. This innovation significantly improves the overall efficiency of the protocol and opens up new possibilities for quantum information transmission. In addition, the protocol effectively saves a large amount of quantum resources while maintaining the same transmission efficiency, which means that we can achieve more efficient resource utilization in quantum communication. The details are as follows.

### 5.1. Extended Protocol (Protocol 2)

In this section, we describe the extended protocol based on the first one, also applicable to smart door locks in IoT scenarios. Similarly, Alice represents the quantum smart door lock, and Bob represents the classical user.

1. Alice prepares 2n single-photon $|+\rangle$ states and sends them to Bob.
2. Once Bob has received all the qubits, the n particles are randomly selected from them to form sequence $S_{1}$, while the remaining particles constitute sequence $S_{2}$. For sequence $S_{1}$, Bob randomly selects either the CTRL operation (which returns the particle to its original state without any additional processing) or the SIFT operation (which measures the particle with the Z-basis, records the measurement, and returns the particle to its original state). For sequence $S_{2}$, Bob performs message encoding with the following encoding rules:
  (a) In such a scenario, should Bob wish to convey the message “00”, he would effectively discard the original particle and prepare a state $|0\rangle$ to return to Alice.
  (b) If Bob wants to convey the message “01”, he would effectively discard the original particle and prepare a state $|1\rangle$ to return to Alice.
  (c) If Bob wishes to convey message “10”, it is necessary to measure the original particle with the Z-basis and record the result (assuming that the measurement is 0), returning an identical state (returning a $|0\rangle$) to Alice.
  (d) If Bob wants to pass on message “11”, he measures the original particle with the Z-basis and records the result (assuming that the measurement is 0), returning an opposite state (returning a $|1\rangle$) to Alice.
  Ultimately, the 2n particles are returned to Alice.
3. Alice receives all the particles and transmits an acknowledgement message to Bob, who then transmits the position of the particles in sequence $S_{1}$, the operation performed on the particles and the measurement results of the SIFT operation. Alice receives and performs eavesdropping detection on the different operations performed by Bob. For particles where Bob performs a CTRL operation, Alice measures them directly with an X-basis, and the result must be $|+\rangle$. The particle selected by Bob for the SIFT operation is directly measured using the Z-basis, and the resulting measurements are then compared with Bob’s own measurements, which should be identical. Alice performs eavesdropping detection based on the results of the above measurements. If the error rate of the detection is higher than a threshold, the communication is abandoned and restarted; otherwise, the process continues to the next step.
4. If there is no eavesdropping, Bob is informed that the channel is secure. Bob informs Alice the location of the remaining particles in which the measurement operation has been performed and the result of the measurement. Alice performs the decoding of the message.
  (a) For particles that are not measured, Alice measures them directly with the Z-basis. If the measurement result is $|0\rangle$, the secret message delivered by Bob is “00”.
  (b) For particles that are not measured, Alice measures them directly with the Z-basis. If the measurement result is $|1\rangle$, the secret message delivered by Bob is “01”.
  (c) In the case of the particles for which Bob performs measurements, Alice conducts measurements with the Z-basis. The measurements should then be compared with those made by Bob. If the results of this comparison are identical, the secret message delivered by Bob is “10”.
  (d) In the case of the particles for which Bob performs measurements, Alice conducts measurements with the Z-basis. The measurements should then be compared with those made by Bob. If the results of this comparison are different, the secret message delivered by Bob is “11”.

**Figure 4:**
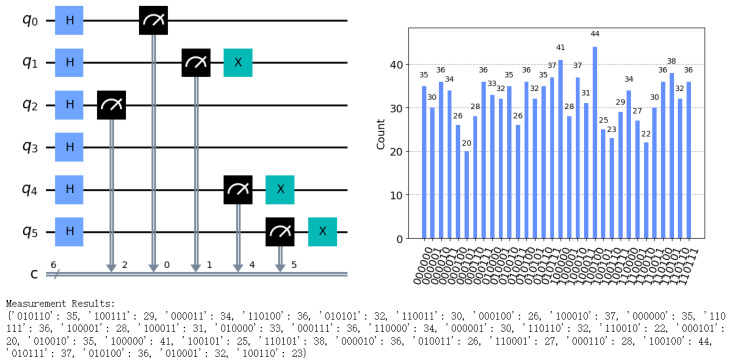
Circuit simulation diagrams and measurement results for extended protocol. The first picture represents the protocol’s circuit simulation diagram. The Hadamard gate (H-gate) is used to create quantum superposition states, the X gate represents a qubit flip operation, and the black arrow means measurement. The remaining picture represents the measurement outcomes for the simulation.

Figure 4 shows the simulated circuit diagram of the communication between Alice and Bob and the results of the measurement of Alice.

### 5.2. Example of Proposed Extended Protocol

**Figure 5:**
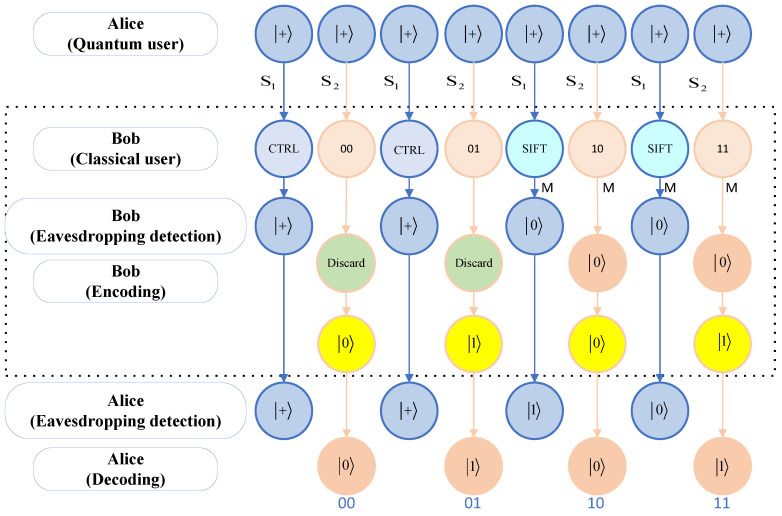
The flowchart of the example.

We give examples of extended protocols, examples of which are as follows. Figure 5 illustrates a process diagram of the extended protocol. The following example illustrates a process within the extended protocol.

1. Assume that Alice prepares 8 states $|+\rangle$ and transmits them to Bob.
2. Bob receives them and randomly selects 4 for the sequence $S_{1}$. Suppose Bob selects the 1st, 3rd, 5th, and 7th particles for the sequence $S_{1}$. Then, the remaining 2nd, 4th, 6th, and 8th particles are sequence $S_{2}$. In this context, it is assumed that Bob performs the operation {CTRL, SIFT, CTRL, SIFT} on the sequence $S_{1}$ for the purpose of eavesdropping detection. If we assume that Bob wishes to deliver the secret message {00, 01, 10, 11}, the first of the four particles of sequence $S_{2}$ is directly discarded, returning a $|0\rangle$; the second particle is directly discarded, returning a $|1\rangle$; the third particle is measured with a Z-basis (assuming that the measurement is 0), and the result is recorded to return an identical state (returning a $|0\rangle$); and the fourth particle is measured with a Z-basis (assuming the measurement is 0), and recording the measurement returns an opposite state (returning a $|1\rangle$). So, for the sequence, Bob returns {$|0\rangle$, $|1\rangle$, $|0\rangle$, $|1\rangle$}.
3. Alice receives 8 particles and sends an acknowledgement to Bob, who tells Alice the location of the particles of sequence $S_{1}$, and the operation performed on the particles, as well as the measurements of the SIFT operation performed, and Alice performs the eavesdropping detection. For particles with direct CTRL operations, Alice measures them directly with an X-basis, and the result should be $|+\rangle$. For the particles that were subjected to the SIFT operation, Alice conducts measurements using the Z-basis and compares the results with those obtained by Bob, which were expected to be identical. If there are no errors in the eavesdropping detection, the process proceeds to the next step. Otherwise, it is necessary to repeat the initial step.
4. Alice informs Bob that the channel is secure, and Bob tells Alice which of the remaining particles have been measured, in this case particles 3 and 4, and that the measurements are {0, 0}. Alice directly measures the particles that were not previously measured (i.e., the 1st and 2nd particles) using the Z-basis. A measurement of “0” for the first particle results in a message of “00” from Bob, and a measurement of “1” for the second particle results in a message of “01” from Bob. For the particles for which Bob performs measurements (i.e., the 3rd and 4th particles), Alice conducts measurements directly with the Z-basis, which results in {0, 1}, and then compares them with Bob’s measurements of {0, 0}, revealing that the third particle has the same result for the comparison, which results in Bob delivering a message of “10”, and the fourth particle has a different result for the comparison, which results in Bob delivering a message of “11”. So, Alice receives the message {00, 01, 10, 11} delivered by Bob.

The security analysis of this extended protocol is similar to that of the initial protocol, which was also found to be secure and twice as efficient.

## 6. Conclusions

This paper presents two semi-quantum secure direct communication protocols based on single-photon technology that address the inefficiencies of existing protocols and are suitable for IoT application scenarios. The preparation of single photons is relatively straightforward, in contrast to the preparation of Bell and GHZ states, which often necessitate more complex interference and entanglement operations. Typically, fewer quantum resources (e.g., qubits) are required for quantum communication using single photons, which can effectively reduce the device complexity and cost. The proposed protocol 1 is concerned with enhancing the efficiency of the protocol, thereby addressing the existing efficiency issue. The extended SQSDC protocol (protocol 2) builds on protocol 1 by enabling a single qubit to transmit two bits of information, resulting in a double efficiency result. Through the combination of quantum communication technology and IoT scenarios, this paper provides a new possibility for IoT communication. This study contributes to the theoretical knowledge base of semi-quantum communication protocols, while also facilitating further development of semi-quantum communication technology through enhancements to protocol design and analysis methods.

## Figures and Tables

**Table 1:** Comparative analysis of SQSDC protocols based on single-photon technology.

Protocol	Efficiency (%)
Zou et al.’s protocol [26]	18.2
Gu et al.’s protocol [27]	9.1
Rong et al.’s protocol 1 [29]	14.3
Rong et al.’s protocol 2 [29]	7.7
Proposed protocol	28.57

**Table 2:** Efficiency comparison.

Protocol	Quantum State	Efficiency (%)
Zou et al.’s protocol [26]	Single-photon	18.2
Zhang et al.’s protocol [35]	Bell states	15.4
Yan et al.’s protocol [36]	Bell states	18.2
Xie et al.’s protocol [37]	Bell states	20
Sun et al.’s protocol 1 [38]	Bell states	10
Sun et al.’s protocol 2 [38]	Bell states	20
Rong et al.’s protocol [30]	Bell states	16.7
Rong et al.’s protocol [30]	GHZ states	12.5
Guo et al.’s protocol [32]	GHZ states	17.65–22.22
Xu et al.’s protocol [33]	Cluster states	18.5
Proposed protocol	Single-photon	28.57

## Data Availability

Data are contained within the article.
